# Counselling for smoking cessation during pregnancy reduces tobacco-specific nitrosamine (NNAL) concentrations: A randomized controlled trial

**DOI:** 10.18332/ejm/99546

**Published:** 2018-11-14

**Authors:** Andriani N. Loukopoulou, Constantine I. Vardavas, George Farmakides, Christos Rosolymos, Charalambos Chrelias, Manolis Tzatzarakis, Aristeidis Tsatsakis, Antonis Myridakis, Maria Lyberi, Panagiotis K. Behrakis

**Affiliations:** 1 General Oncology Hospital of Kifissia ‘Agioi Anargyroi’, Athens, Greece; 2 George D. Behrakis Research Lab, Hellenic Cancer Society, Athens, Greece; 3 Institute of Public Health, American College of Greece, Athens, Greece; 4 General Maternity Hospital Helena Venizelou, Athens, Greece; 5 School of Medicine, National and Kapodistrian University of Athens, Athens, Greece; 6 Maternity Unit, Attikon Hospital, Athens, Greece; 7 Laboratory of Toxicology, School of Medicine, University of Crete, Heraklion, Greece; 8 Environmental Chemical Processes Laboratory (ECPL), Department of Chemistry, University of Crete, Heraklion, Greece; 9 Integrative Systems Medicine and Digestive Disease, Department of Surgery and Cancer, Faculty of Medicine, Imperial College London, United Kingdom

**Keywords:** smoking cessation, pregnancy, cognitivebehavioural, interventions, tobacco-specific carcinogen, NNAL

## Abstract

**INTRODUCTION:**

Smoking cessation during pregnancy is beneficial to both the mother and child. Our objective was to assess if an intensive smoking cessation intervention for pregnant women increases: a) rates of smoking cessation, and b) reduces exposure to tobacco-specific carcinogens during pregnancy.

**METHODS:**

A two-group single-blinded parallel randomized controlled trial (RCT) was conducted involving 84 pregnant smokers in either a high intensity (n=42) or minimal contact control group (n=42). Women assigned to the high intensity smoking cessation intervention group received a single 30-minute behavioural counselling session and a tailored self-help booklet. The primary outcome measures were: 7-day point prevalence abstinence measured by selfreport and urine cotinine levels, and maternal tobacco specific carcinogens nitrosamine (NNAL) urine concentrations assessed at 32 weeks of gestation.

**RESULTS:**

A significantly greater percentage of pregnant smokers quit smoking in the high intensity group compared to the low intensity control group (45.2% vs 21.4%; p=0.001). A significant decrease in urine cotinine concentrations was documented in the experimental group (-140.74 ± 361.70 ng/mL; p=0.004), with no significant decrease documented in the control group. A significant decrease in NNAL levels was also documented in the experimental group (158.17 ± 145.03 pg/mL before, 86.43 ± 112.54 pg/mL after; p=0.032) with no significant changes in the control group.

**CONCLUSIONS:**

The high intensity intervention tested resulted in significantly greater cessation rates. Intensive smoking cessation interventions can be effective in reducing fetal exposure to NNAL. This is the first trial to report on NNAL tobacco-specific carcinogen concentrations before and after an intervention for smoking cessation during pregnancy.

**TRIAL REGISTRATION:**

ClinicalTrials.gov Identifier: NCT01210118.

**ABBREVIATIONS:**

5Αs: ask, advise, asses, assist, arrange; GHQ: general health questionnaire; ANOVA: analysis of variance; RCT: randomized control trials; NNAL: 4-(methylnitrosamino)-1-(3-pyridyl)-1-butanol.

## INTRODUCTION

Maternal smoking during pregnancy is associated with multiple adverse outcomes, including increased perinatal mortality rate, premature labour, low birth weight and fetal growth restriction, and health effects that may extend into childhood^1-4^. Maternal smoking during pregnancy may also expose the foetus to tobacco specific carcinogens, such as 4-(methylnitrosamino)-1-(3-pyridyl)-1-butanol (NNAL) and pose a threat of cancer in the foetus and in the newborn’s future life; documented by NNAL in the urine of newborns of mothers who smoked, but not in the urine of neonates of non-smoking mothers^5,6^.

On the contrary, smoking cessation during early pregnancy may ameliorate the negative outcomes^7-10^. Smoking cessation during pregnancy is usually influenced by several factors that include personal, family, educational and social characteristics, such as age, educational level, employment, marital status, stress and the partner’s smoking status^11-13^. Therefore, smoking cessation in pregnancy is of significant importance, and the new social role as mothers, make pregnancy a ‘teachable moment’, as a women’s receptivity toward smoking cessation messages is increased^14^. To maximize the value of this ‘teachable moment’ it is important to provide expectant mothers with evidencebased smoking cessation interventions. Behavioural counselling delivered with sufficient intensity (minimum of 15 minutes) has been shown to significantly increase rates of smoking abstinence^15,16^. However, tobacco-use treatment is infrequently delivered in the obstetrics setting. Furthermore, no published studies have examined if cessation during pregnancy results in reduced concentrations of tobaccospecific carcinogens. As such, the purpose of the Maternal Smoking Cessation during Pregnancy (M-SCOPE) study was to assess if an intensive smoking cessation intervention for pregnant women increases: a) rates of smoking cessation, and b) reduces exposure to tobacco-specific carcinogens during pregnancy compared to a low intensity control group. Secondary exploratory outcomes included: birth outcomes (birth weight, prematurity of birth) and complications during pregnancy.

## METHODS

### Study design

The M-SCOPE study was a two-group, single blind, parallel randomized controlled trial (RCT) that compared a high intensity intervention to a low intensity control group among pregnant women recruited from two hospitals in Athens, Greece. Follow-up measurement occurred in week 32 of gestation. The complete study protocol, design and methodological approach are described in detail elsewhere^17^. Ethical approval was provided by the Biomedical Ethics Committee of the Faculty of Medicine of the National and Kapodistrian University of Athens (Protocol approval number: 4568/07-01-08) and the Ethics Committee of each participating hospital: Peripheral General Maternity Hospital ‘Elena Venizelos’ (Protocol approval number: 137/04-10-07) and the Maternity Unit of the ‘Attikon’ University Hospital in Athens (Protocol approval number: 287/30-07-09). The trial was registered on ClinicalTrials.gov (Identifier: NCT01210118).

### Inclusion/exclusion criteria

Inclusion criteria of participants were: a) currently pregnant, b) current cigarette use of >5 cigarettes over the past 7 days, and c) age >18 years. Exclusion criteria were: a) a gestational age less than 24 weeks at the time of enrolment, b) limited or no telephone access, c) not planning to live at the same address for the next year, d) unable to read and/or speak Greek fluently, e) current alcohol or substance abuse (defined as strong cravings for alcohol, inability to limit drinking, continued use of alcohol despite the repeated problems)^18^, and f) current depression (according to the Greek validated version of the Goldberg General Health Questionnaire (GHQ)^19,20^.

### Procedures

Recruitment took place from November 2009 to February 2012. The first contact (the baseline assessment), took place before the 24th week of gestation. Written informed consent was obtained from all participants who completed a survey at baseline to document demographic and smoking related variables. Participants were randomly assigned to one of the two intervention arms using a computer random number generator, placing random number assignments in an opaque envelope that was prepared by a third party and opened only after participants had provided informed consent concealed allocation. Patients were blinded to their study assignment group (single blind). Participants returned to hospital for follow-up assessment in week 32 of gestation. At the baseline and 32 weeks follow-up visit, each participant was requested to provide a urine sample for nicotine/cotinine and NNAL analysis. Urine cotinine and nicotine concentrations were assessed through liquid chromatography mass spectrometry (LC/MS) analysis. The chromatographic separation was achieved using a Thermo Finnigan Surveyor LC system (Thermo Finnigan, San Jose, USA), equipped with a Gemini C18 (3 μm, 100 mm × 2 mm) analytical column by Phenomenex (Torrance, USA). The mass detection was achieved with a TSQ Quantum triple quadrupole with ESI source operated in positive mode (Thermo Finnigan, San Jose, USA). The system was controlled by the Xcalibur software, which was also used for data acquisition and analysis^21,22,24,25^. Urine NNAL was analysed as previously described^17,25^. The cut-off noted by Melvin et al. and Spierto et al. of ≤80 ng/mL for urinary cotinine was used for the biochemical validation of smoking abstinence^15,23^. Following childbirth, third assessment occurred during which data on birth outcomes and pregnancy complications were collected.

### Intervention comparators

Control group participants received a minimal contact intervention, which included face-to-face communication for 5 minutes and the provision of brief advice and a leaflet on smoking cessation during pregnancy. Experimental group participants received a higher intensity intervention, which included: a single 30-minute face-to-face cognitivebehavioural counselling session based on the ‘5Αs’ (ask, advise, asses, assist, arrange) model^24,26^, delivered by a specially trained registered nurse. During the counselling session the participating women received a self-help manual, specifically tailored for smoking cessation during pregnancy for Greek women. The self-help manual was divided into four parts. The first part summarized the key points in regard to the effects of smoking during pregnancy on the foetus, but also of the gains acquired through smoking cessation, the benefits for maternal health and how to prevent relapse and stay smoke-free. A questions-and-answers list was included based on common queries, such as the best time to quit, breast-feeding issues, weight gain, etc. The second part of the self-help manual aimed to prepare the pregnant woman to quit by providing practical solutions for handling cravings and nervousness, the importance of the involvement of the partner and the woman’s social network. The third section of the manual was about setting a smoking cessation date. Some general practical suggestions were included as well as some special suggestions for the quit-date. The booklet emphasized the importance of the pregnant woman’s will to remain abstinent. On the last page of the self-help manual was a visual on the health benefits of quitting to mother and foetus.

### Outcome measures

The primary outcome measures assessed were the participant’s 7-day point prevalence smoking status during the 32nd week of gestation, changes in urinary nicotine and cotinine concentrations, as well as urinary NNAL levels. Secondary exploratory outcomes included: birth outcomes (birth weight, prematurity of birth) and complications during pregnancy, which were assessed using participant self-report and medical record verification.

### Recruitment

**Figure 1:**
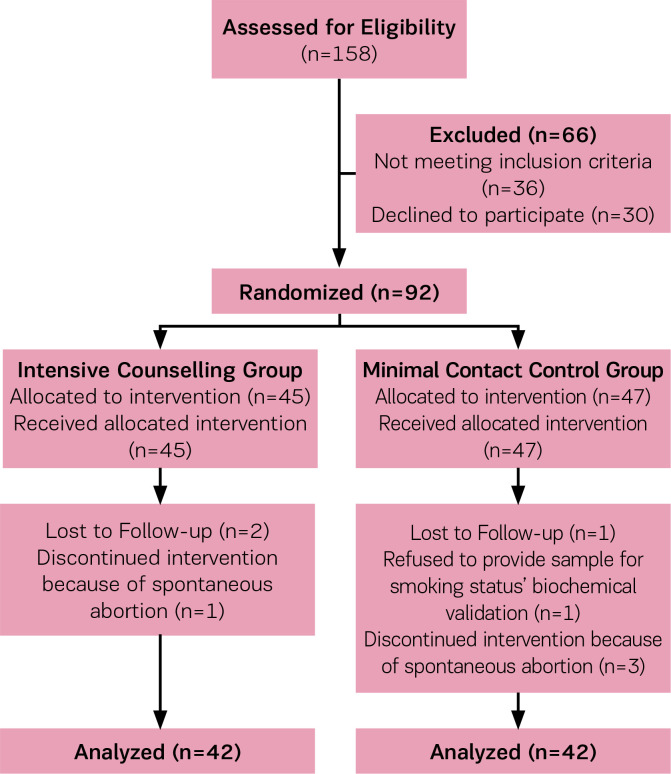
Flow diagram of M-SCOPE Study

A total of 746 pregnant women were screened, 541 were not eligible to participate because they reported themselves as non-smokers, and 47 were at >24 weeks of gestation at screening. Of the 158 pregnant smokers who remained, 36 pregnant smokers were ineligible for the following reasons: suffered from depression according to the GHQ (n=2), used drug substances (n=1), using methadone (n=1), reported spontaneous abortions (n=9), reported that they had already quit smoking before arranging the consultation (n=20), changed telephone number (n=1), changed maternity hospital (n=2), while 30 pregnant smokers declined to participate in the research. Therefore, the final sample size comprised 92 pregnant smokers who were randomly assigned either to the control group (n=47) or the intervention group (n=45). During the study, four of the enrolled pregnant women were excluded because of miscarriage while four withdrew. Therefore, 84 pregnant smokers completed this study and were included in the analysis. The study flow diagram is presented as Figure 1.

### Statistical analyses

The descriptive data of this study are presented as mean ± standard deviation for continuous variables and as percentages for categorical variables. General descriptive statistics were used to describe the participant’s demographic characteristics, smoking habits, smoking status and exposure to secondhand smoke. Non-parametric tests were used, such as Pearson’s χ^2^ test and Fisher’s exact test for comparison of proportions, while Student’s t-test or the non-parametric test Mann-Whitney were used for the comparison of quantitative variables between the two intervention groups. The Wilcoxon signed-test was used for the comparison of urine nicotine and cotinine between the pre- and post-measurements. The analysis of variance for repeated measurements (ANOVA) was used to check the differences in the measurements between the groups. Logistic regression analyses were performed. All statistical tests were two-sided, and a p<0.05 was considered as statistically significant. Data analysis was performed using a statistical package for the Social Sciences (PASW)^27^ version 18.

## RESULTS

The study population demographic characteristics at baseline are presented in Table 1. There were no statistically significant differences between control and intervention group participants for demographic characteristics or smoking history. Notably, the majority of both intervention and control group participants had low nicotine dependence during pregnancy according the Fagerström Test for Nicotine Dependence, (54.8 % in control group and 64.3% in intervention group).

**Table 1:** Participant characteristics

*Variable*	*Response*	*Control % (N)*	*Intervention % (N)*	*p*
**Week of gestation at enrolment (Mean ± SD)**		19.5 ± 5	15.7 ± 6,4	0.002+
		21.5 (17–24)	17.5 (8–21)	
**Age (Mean ± SD)**		32.4 ± 4.5	31.4 ± 5.9	0.399**
**Nationality**	Other	4.8 (2)	4.8 (2)	1.000*
	Greek	95.2 (40)	95.2 (40)	
**Educational level**	Low/Medium	61.9 (26)	57.1 (24)	0.657
	High	38.1 (16)	42.9 (18)	
**Marital status**	Engaged	9.5 (4)	26.2 (11)	0.046
	Married	90.5 (38)	73.8 (31)	
**Current work status**	Public employee	21.4 (9)	7.1 (3)	0.171
	Private employee	47.6 (20)	42.9 (18)	
	Free lancer	9.5 (4)	14.3 (6)	
	Unemployed/ Household/ Student	21.4 (9)	35. 7 (15)	
**Age of smoking initiation (Mean ± SD)**		18.1 ± 3.5	17.2 ± 2.5	0.393+
		17 (16–20)	17 (16–19)	
**Years of smoking (Mean ± SD)**		14.3 ± 5.5	14 ± 5.4	0.858**
**Number of cigarettes smoked before pregnancy (Mean ± SD)**		22.1 ± 9.7	19.7 ± 8,6	0.414+
		20 (15–30)	20 (15–25)	
**Number of cigarettes smoked during pregnancy (Mean ± SD)**		6.7 ± 5.1	6.7 ± 5,3	0.669+
		6 (4–7)	5 (3– 9)	
**Prior attempts to quit smoking**	No	40.5 (17)	42.9 (18)	0.825
	Yes	59.5 (25)	57.1 (24)	
**Number of quit attempts (Mean ± SD)**		1.1 ± 0.3	1.8 ± 2	0.197+
		1 (1–1)	1 (1–1.5)	
**Duration of prior quit attempts in weeks (Mean ± SD)**		23.3 ± 27.8	21.3 ± 32.3	0.431+
		12 (4–32)	8 (4–28)	
**Is your partner a smoker?**	No	35.7 (15)	23.8 (10)	0.233
	Yes	64.3 (27)	76.2 (32)	0.866+
**Number of cigarettes smoked by partner daily (Mean ± SD)**		23 ± 13.9	22.2 ± 13, 6	
		20 (10–40)	20 (10–30)	0.195
**Low Nicotine Dependence (Fagerström Test for Nicotine Dependence)**		54.8 (23)	64.3 (27)	

### Self-reported changes in smoking

Self-reported changes in smoking status are presented in Table 2. A significantly greater percentage of pregnant smokers quit smoking in the intervention group compared to the control group (45.2 % vs 21.4%, p=0.001). Furthermore, the percentage of mothers who reduced smoking during pregnancy was higher among intervention group participants in comparison to the control group participants (35.7% vs 23.8% p=0.09).

**Table 2:** Participants self reported smoking status by group at follow-up

*Outcome measure*		*Control % (N)*	*Intervention % (N)*	p*
Self-reported smoking status	Continuation	59.5 (25)	35.7 (15)	0.090
	Reduction	23.8 (10)	35.7 (15)	
	Cessation	16.7 (7)	28.6 (12)	
Have you reduce or quit smoking?	No	59.5 (25)	35.7 (15)	0.029
	Yes	40.5 (17)	64.3(27)	

* Pearson’s χ^2^ test.

### Changes in urinary cotinine/nicotine and NNAL

**Figure 2:**
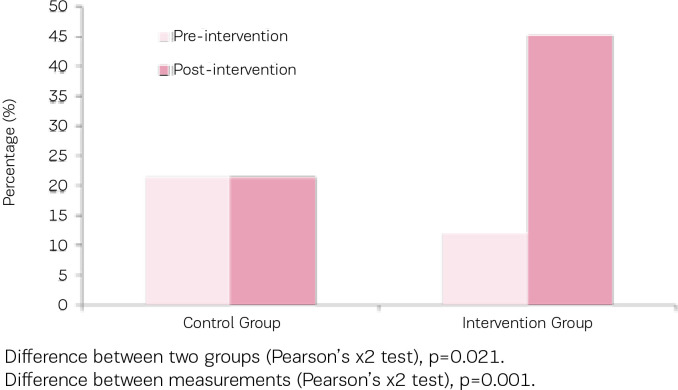
Percentage of participants who quit smoking in both groups (urine cotinine ≤80 ng/Ml after intervention)

As indicated in Table 3, concentrations of urinary cotinine of both groups were at similar levels before the intervention, however following the intervention concentrations of urinary cotinine showed a statistically significant decrease in the intervention group (mean change: -140.7 ± 361.7 ng/ mL; p=0.004), a reduction greater than that noted within the control group (mean change: -82.1 ± 485.7 ng/mL; p=0.228). Similarly, the urinary nicotine concentrations in both groups were similar at baseline, however they significantly decreased in the intervention group (mean change: -190.1 ± 620 ng/mL; p=0.005), but not in the control group (Figure 2).

**Table 3:** Urine nicotine and cotinine concentrations of study participants before and after intervention by group

*Bio-chemical measure*	*Pre-Intervention Mean ± SD*	*Post-Intervention Mean ± SD*	p*
**Urine nicotine**			
Control group	444.5 ± 760.6 ng/mL	678.9 ± 1467.2 ng/mL	0.812
	443.7 ± 666.9 ng/mL	253.6 ± 532.3 ng/mL	
**Urinary Cotinine**			
Control group	561.7 ± 663,9 ng/mL	479.6 ± 563.4 ng/mL	0.228
Intervention group	452.7 ± 516.9 ng/mL	311.9 ± 490.1 ng/mL	0.004
**Urinary NNAL**			
Control group	106.35 ± 62.9 pg/mL	132.30 ± 100.6 pg/mL	0.201
Intervention group	158.17 ± 145.03 pg/mL	86.43 ± 112.53 pg/mL	0.032

* Wilcoxon test

A significant decrease in NNAL levels was also noted in the intervention group (158.1 ± 145.0 pg/mL vs 86.4 ± 112.5 pg/mL; p=0.032), in contrast, control group levels increased after the intervention (106.35 ± 62.85 pg/mL vs 132.30 ± 100.6).

### Perinatal outcomes

Perinatal outcomes were examined on an exploratory basis, the results of which are shown in Table 4. Overall, no statistically significant differences between smoking cessation and perinatal outcomes were documented in our underpowered analysis.

**Table 4:** Weeks of pregnancy until childbirth (Mean ± SD)

*Variables*		*Smoking status (cotinine concentrations)*		*p*
		*Non-smoker (≤80 ng/mL) % (N)*	*Smoker (>80 ng/mL) % (N)*	
Birth weight in grams (Mean ± SD)		3241.2 ± 444.5 3235 (2975–3582.5)	3056.4 ± 498.8 3000 (2750–3420)	0.100***
Premature birth	No	46.4 (13)	53.6 (15)	0.072**
	Yes	26.8 (15)	73.2 (41)	
Prematurity of birth in days (Mean ± SD)		9.6 ± 7.8 7 (4–11)	13.2 ± 9.9 10.5 (7–15.5)	0.208***
Weeks of pregnancy until childbirth (Mean ± SD)		39.2 ± 1.1 40 (39–40)	38.4 ± 1.9 39 (38–40)	0.038+*
Complications during pregnancy	No	32.9 (23)	67.1 (47)	1.000**
	Yes	35.7 (5)	64.3 (9)	
Complications at labour	No	35.4 (28)	64.6 (51)	0.164**
	Yes	0.0 (0)	100.0 (5)	

* Pearson’s χ^2^ test

** Fisher’s exact test

*** Student’s t-test

+ Mann-Whitney test.

## DISCUSSION

Our results demonstrate that an intensive counselling intervention for smoking cessation can be effective in aiding smoking cessation during pregnancy compared to a minimal contact intervention. Our findings are in agreement with the results of a systematic review^28^ of nineteen interventions that rate the effectiveness of various smoking cessation interventions during pregnancy, which reported rates of smoking abstinence of 26.5–47%. Our findings are also consistent with the results of a large systematic review and a meta-analysis that evaluated the most effective counselling interventions for smoking cessation during pregnancy. A recent 2017 Cochrane Review by Chamberlain et al. examining psychosocial interventions for pregnant women who smoked found high quality evidence that counselling interventions significantly increased smoking abstinence compared with usual care (30 studies; average RR=1.44, 95% CI: 1.19–1.73) and resulted in important reductions in adverse pregnancy outcomes^16^. A meta-analysis by Melvin et al. also highlighted the importance of sufficient intensity and duration of a cognitive-behavioural intervention delivered to pregnant women noting interventions should last about 15 minutes and be accompanied by printed material^15^. In addition, previous research has indicated that the provision of self-help materials to pregnant women may provide a modest but significant effect (RR=1.21, 95% CI: 1.05–1.39), while research has indicated that materials that were specifically tailored for smoking cessation during pregnancy were more effective than general smoking cessation materials (RR=1.31, 95% CI: 1.20–1.42)^15^. The intervention tested in the M-SCOPE study was based in available evidence and included a cognitive-behavioural intervention lasting 30 minutes and the provision of printed self-help manual specifically tailored for smoking cessation during pregnancy^17^.

The results of the present study indicate that the prenatal exposure to tobacco-specific carcinogens was directly affected by the implementation of the intensive smoking cessation intervention that led to a significant reduction in NNAL concentrations in the urine of pregnant smokers who quit smoking. To the best of our knowledge, there have been no reports on the analysis of carcinogens or their metabolites in the urine from pregnant smokers who participated in a smoking cessation study. Thus, this is the first clinical trial that studies the tobacco-specific nitrosamine NNAL concentrations before and after an intervention for smoking cessation during pregnancy, as well as the levels of urine nicotine and cotinine concentrations. These results are similar to findings of an observational study by Vardavas et al. which found pregnant smokers have mean urinary NNAL concentrations of 0.612 pmol/mL, compared to the 0.100 pmol/mL of ex-smokers and 0.0795 pmol/mL of non-smokers exposed to secondhand smoke^29^. Generally, urinary NNAL levels were well correlated with urinary cotinine levels as reported by other authors^30,31^. The adverse effects of NNAL concentration of pregnant smokers to the unborn feotus have been described by Florek et al. who found that NNAL, present in the urine of pregnant women who smoke tobacco, crosses the placenta and poses a threat of cancer in the fetus and newborn’s future life. In other words, unborn children of women who smoke during pregnancy are exposed to toxic constituents of tobacco smoke that cross the placental barrier^5^. According to Lackmann et al. higher urinary NNAL levels, averaging 29.3 pg/mL (95% CI: 17.3–41.8) were found in newborns of mothers who smoked during pregnancy^6^. Exposure to the intensive counselling intervention tested in this study reduced fetal exposure to tobacco specific nitrosamines eliminating the threat of cancer in their future life.

Infants’ birth weight among participants who quit smoking tended to be higher (3241.2 ± 444.5 g), compared to those who continued smoking (3056.4 ± 498.8 g). The mean difference in birth weight between the infants of participants who quit smoking and the infants of those who continued smoking was 235 g. This result is consistent with previous studies, in which women who smoked during pregnancy had almost 150–250 g lower birth weight infants compared to infants of non-smokers^16,32^.

Our study has important implications to practice. First the study demonstrates the intervention tested is both efficacious and feasible to implement in the obstetrics setting. Given the low treatment rates in obstetrics and gynecology practice and other settings in which care is provided to pregnant women, supporting the introduction of such evidence-based smoking cessation counseling services as a standard-ofcare should be a priority. The intensive intervention tested involved a single 30-minute counseling session considered low cost, in particular in view of the outcomes that resulted. Additionally, in order to eliminate the effects of tobacco use on women and increase an effective perinatal smoking abstinence, the focus should be extended to all women of reproductive age in order to support cessation prior to becoming pregnant. Given the risks of secondhand exposure to the foetus, consideration should be given to extending smoking cessation services to include the whole family^29^.

### Study strengths and limitations

A limitation of this study might be the different week of gestation at enrolment between control and intervention group, 19.5 ± 5.0 and 15.7 ± 6.3, respectively. As week of gestation at enrolment might be considered one of the indicators for smoking cessation, it might constitute a source of potential bias. Although the mean week of gestation at enrolment between the two groups was different, this was not found to have affected the quit rates. Another possible limitation of this study is that pregnant smokers might have misreported baseline cigarette consumption, as smoking during pregnancy is not socially accepted. Moreover, the results of this study may not be generalizable to other countries due to different cultural backgrounds. Our study has significant strengths, which include its robust RCT study design, the biochemical validation of self-reported smoking status and analysis of tobacco-specific nitrosamine NNAL concentrations before and after an intervention for smoking cessation during pregnancy.

## CONCLUSIONS

Our results demonstrate the importance of higher intensity counselling interventions for smoking cessation during pregnancy and the feasibility of successfully implementing such interventions in clinical practice, especially in Greece where no organized smoking cessation programmes for pregnant smokers are at present provided. Moreover, the remarkable decrease of 45% of tobacco specific carcinogens during pregnancy, and after the intensive intervention took place, indicates that intensive smoking cessation interventions can be effective in reducing fetal exposure to tobacco specific nitrosamines, with direct health benefits for both mother and foetus.

## CONFLICTS OF INTEREST

Authors have completed and submitted the ICMJE Form for Disclosure of Potential Conflicts of Interest and none was reported.
